# Affect Is at the Heart of Norm Psychology: Commentary on Heyes, “Rethinking Norm Psychology”

**DOI:** 10.1177/17456916231187390

**Published:** 2023-08-25

**Authors:** Jonathan Birch

**Affiliations:** Department of Philosophy, Logic and Scientific Method, London School of Economics and Political Science

Heyes offers a strikingly minimalist picture of the implicit (“Type 1”) processes involved in norm compliance and enforcement. Like rats and many other animals, people are good at model-free reinforcement learning (and Heyes seems to say, although I would welcome clarification on this, that the type of reinforcement learning involved in implicit processing is model-free). They also like the feeling of doing an action if they have learned an association between that action and reward. People like predictable environments and dislike surprises.

In short, the picture is one on which the implicit side of norm psychology is just not very special. The “special” part of norm psychology, the part that may well be distinctively hominin, is to be located elsewhere, in explicit (“Type 2”) normative reasoning and in the verbal commentary people offer on each other’s behavior. This, like other varieties of explicit reasoning, is plausibly culturally inherited and culturally evolved.

Although I agree about the importance of culture on the explicit side (for more on that, see Birch & Heyes, 2021), I have trouble signing up to such a minimalist picture of the implicit side. But what is missing? What is not explainable by a mix of model-free reinforcement learning and an affinity for predictable environments? One source of unease is the following sort of example:

***Contract:*** I’m talking to a colleague, and I know their contract is going to be terminated. Everything will be much easier for me if I can avoid telling them this. I keep telling myself “Don’t go there!” They don’t know I know, they will soon hear it from a formal letter, and I don’t know how they will react when they hear. Yet the affective pressure created by internalized norms of collegiality and honesty gradually builds up until I feel I just *have* to tell them.

What explains this “affective pressure”? Not my affinity for predictable environments because following the norms of collegiality and honesty will make an everyday situation enormously more unpredictable. My selfish preference for predictability is part of what is overridden by the affective pressure. And model-free reinforcement learning seems to provide a similarly inadequate explanation. I get no reward at all from saying “you’re fired” or from causing upset to others; what I expect to follow is intense discomfort.

So given this example, I seem to be outside the range of responses that the implicit processes on the table can explain. Yet I am not in the territory of explicit processing either. In fact, this is a case of dissonance between explicit and implicit processing. My explicit reasoning is telling me “Don’t go there!” but the norms I have internalized drag me around to a different course.

For all I know, the sort of dissonance present in “Contract” could be a distinctively “WEIRD” (Western, educated, industrialized, rich, and democratic) experience, not a human universal. Yet it does not need to be universal to generate a problem for Heyes’s minimalism. If there is even one culture in which implicit processes often generate affective pressure toward following a norm even though model-free reinforcement learning, aversion to unpredictability, and explicit reasoning are all pushing the other way, then the minimalist picture has fallen short, and some other mechanisms are at least sometimes involved.

Heyes’s article includes the seeds of an interesting response:

Explicit processes can also become implicit. Like driving a car, patterns of normative thought that were once deliberative—conscious, effortful—can become automatic with intensive practice. . . . Rules such as *Do not tamper with nature* and *Acts are worse than omissions* can “go underground,” becoming “heuristics” or “intuitions” with a pervasive influence on behavior that is unexplained by . . . , or at odds with, the actor’s normative commentary. (p. 28)

The analogy with driving points to a close relationship between the psychology of norms and the psychology of skills, and I argued elsewhere that there is indeed an evolutionarily deep connection here (Birch, 2021a, 2021b). Analogies between norms and skills have a long history in philosophy, starting with Aristotle, and they deserve more attention in psychology.

Could it be that in “Contract,” I have learned to be a good colleague in the way I once learned to drive, with explicit instruction gradually giving way to well-tuned intuitions? A problem is that the role of affective pressure is very different in the two cases. When one has a practical skill, one can choose to “switch off” one’s internalized standards. Skilled tennis players can play deliberately amateurishly with their children rather than bombarding them with 120-mph serves. By contrast, social norms exert a grip that persists even when people intend to disobey them. I can set myself the goal of avoiding conflict by telling half-truths to my colleagues, but my affective system will not stand for it.

Something must be added to the minimalist picture, in my view. One alternative approach is that of Peter Railton, who has developed a view on which the affective system is continuously engaged in prospective, evaluative simulation of possible courses of action, all occurring implicitly (Railton, 2014, 2017, 2021). In “Contract,” on this view, I implicitly simulate that my colleague is at risk of being crushed by the formal letter, and I simulate that I can mitigate the risk by breaking the news sensitively. My explicit thoughts take a completely different direction, but my implicit simulations do their work independently, manifesting as affective pressure. The norms I have internalized are implicitly encoded in the value weightings the simulations give to different types of risk.

One might call Railton’s picture “maximalist” because it is attributing so much sophistication on the implicit side. Intermediate positions are possible, and more work exploring that middle ground would be helpful. For now, my contention is that a framework for guiding future research into the psychology of norms needs to give high priority to understanding (a) the connection between norms and skills and (b) the central role of affect in our normative lives.

## Transparency

*Action Editor:* Daniel Kelly

*Editor:* Interim Editorial Panel
